# Women with a predisposition for diabetes have an increased risk of pregnancy complications, especially in combination with pregestational overweight

**DOI:** 10.1186/s12884-020-2741-5

**Published:** 2020-02-03

**Authors:** Ulrika Moll, Håkan Olsson, Mona Landin-Olsson

**Affiliations:** 1 Department of Endocrinology, Lasarettsgatan 15, Skane University Hospital, S-221 85 Lund, Sweden; 2 Department of Clinical Sciences, Lund University, Lund, Sweden; 3 Departments of Oncology & Pathology and Cancer Epidemiology, Skane University Hospital, Lund, Sweden

**Keywords:** Gestational diabetes, Overweight, Pregnancy outcome, Caesarean section, Macrosomia

## Abstract

**Background:**

Overweight and gestational diabetes are risk factors for pregnancy complications. We hypothesized that the metabolic impact of overweight on pregnancy outcome, would be different if it was combined with a predisposition for diabetes. The aim of this study was to compare the outcome of pregnancies in women with diabetes diagnosed later in life, to the outcome of pregnancies of women who did not develop diabetes.

**Methods:**

Women in a population-based cohort who also were registered in the Swedish Medical Birth Registry (*n* = 4738) were included. A predisposition for diabetes (GDM or diabetes after pregnancy) was found in 455 pregnancies. The number of pregnancies with maternal BMI ≥ 25 kg/m^2^ and without diabetes were 2466, and in 10,405 pregnancies the mother had a BMI < 25 kg/m^2^ without diabetes at any time. Maternal BMI, gestational length, gestational weight gain, frequency of caesarean section, infant birth weight, frequency of large for gestational age (LGA) and Apgar score were retrospectively compared.

**Results:**

Pregnancies with normal maternal BMI ≤25 kg/m^2,^ with predisposition for diabetes had a higher frequency of LGA (11.6% vs. 2.9%; *p* < 0.001), a higher frequency of macrosomia (28.6% vs. 17.6%; p < 0.001), and a shorter gestational length (39.7 vs. 40 weeks; *p* = 0.08) when compared to pregnancies in women without a predisposition for diabetes. In addition, pregnancies with both maternal predisposition for diabetes and BMI ≥ 25 kg/m^2^ there was a higher frequency of LGA (23.3% vs. 7.1%; *p* < 0.001), caesarean section (24.0% vs. 14.9%, *p* = 0.031) compared to pregnancies in women who were only overweight. A predisposition for diabetes significantly increases the risk of macrosomia (OR1.5; 95% CI 1.07–2.15; *p* = 0.02).

**Conclusions:**

In pregnancy, there is an increased frequency of LGA, macrosomia and caesarean section if the woman has a predisposition for diabetes. The frequency of overweight young women is increasing, and it is urgent to identify pregnant women with a predisposition to diabetes. How to distinguish the women with the highest risk for adverse pregnancy outcome and the highest risk of future disease, remains to be studied.

## Background

Maternal overweight and obesity are risk factors for several complications during pregnancy and delivery [1, 2]. This leads to increased foetal growth, increased risk for large for gestational age (LGA) infants and for caesarean section [3–9]. A combination of a high maternal Body Mass Index (BMI) and a high gestational weight gain (GWG) further increases the risk for caesarean section and LGA births. A low GWG among obese women is beneficial and reduces the risks of complications during pregnancy and delivery [10]. Therefore the Institute of Medicine (IOM) have issued recommendations regarding restricted weight gain during pregnancy [11].

In our cohort we previously found that obesity and overweight at the start of pregnancy increased the risk of obesity as well as diabetes and cardiovascular disease 10–27 years after pregnancy. However, we found that in women with a high gestational weight gain there was no significant risk of future diabetes or cardiovascular disease [12].

It is well known that gestational diabetes (GDM) can result in diabetic fetopathy with an increased risk for caesarean section and postnatal complications [13, 14]. Additionally, there is an increased risk of stillbirth before the clinical onset of diabetes [15]. There is increasing evidence that slightly elevated glucose even below diagnostic levels for diabetes and IGT, could be harmful and increase the risk of LGA-infants and caesarean sections [16–18]. The question has been raised whether GWG, being overweight before pregnancy and GDM are equal risk factors for LGA births and complications during delivery. A study of a Swedish cohort showed that both normal weight women with GDM and overweight women without GDM had similar risks of LGA-infants and caesarean section [19]. Heude et al. showed that the risk of LGA births increased in parallel with increasing prepregnancy BMI but did not correlate with GWG [20]. On the contrary, another study showed that both a high GWG and a high prepregnancy BMI increased the risk of delivering LGA infants [6]. The risk was even higher in women who at the same time have GDM [21].

Consequently, there seems to be a synergistic effect for negative pregnancy outcome between obesity and GDM. Sovio et al. showed that obesity or GDM alone resulted in a doubled risk of a high abdominal circumference in the infant at the 28th week of gestation. Furthermore, a combination of both GDM and obesity resulted in a fourfold risk of LGA-infant at birth [22].

Our aim was to study the effect of having a predisposition for diabetes (defined as onset of diabetes later in life) and overweight, alone or in combination, on pregnancy outcome.

## Methods

### Study population

A cohort of 29,488 women, in the ages 25–65 years, representing every eighth woman (12.5%) in the Population Register, in the southern region of Sweden was established in 1990 to study malignant melanoma. The MISS (Melanoma in southern Sweden) cohort have been followed since then [23]. At the time of cohort establishment, the women answered a questionnaire regarding social status, previous illnesses, medication, weight and height. In a follow-up-study of the same cohort 10 years later, in which 23,524 women participated, a more extensive questionnaire was used, with additional questions regarding diseases, medication and life style. The women were then between 35 and 75 years old. Based on self-reported answers we characterized the woman to have diabetes mellitus if she was using any prescribed anti diabetic medication or if she self-reported diabetes (without further specification) among current diseases. In the follow up study we identified 808 (3.4%) women with diabetes mellitus, which is an expected frequency of diabetes in a Swedish population.

After excluding women with Type 1 diabetes we identified 14,811 pregnancies in the Swedish Medical Birth Registry (SMBR) between 1973 and 2005 related to the women participating in the MISS cohort. SMBR started in 1973 and approximately 100,000 births per year in Sweden have been registered since then and the dropout rate is only 0.5–3% [24]. The register contains data concerning maternal characteristics during pregnancy, delivery and postnatal data regarding the infant.

The reported data in SMBR regarding GDM is not complete. During these years there was no general screening for GDM in southern Sweden. The women attended many different prenatal care units, with different routines regarding screening for hyperglycemia during pregnancy. Between 1973 and 1989 diabetes was only registered as “yes” or “no” depending on if there was a registered ICD-code or not. The more recent registration system which registers diabetes as “chronic” or “transitory” gives a higher registered frequency. Diabetes as current/chronic disease i.e. diabetes existing prior to pregnancy seems adequately registered (*n* = 45). This gives a prevalence of 0.9% which is an expected prevalence of diabetes in a Swedish female pregnant population. We considered these women mainly to be patients with type 1 diabetes and they are not included in the analyses.

By merging the MISS cohort and SMBR, we could retrospectively study the pregnancy and the delivery in relation to the woman’s BMI (Body Mass index) at the start of pregnancy and in relation to later development of diabetes.

Pregnancies before the start of SMBR in 1973 were not included in the study. Data regarding BMI for the first registered pregnancy were found for 4738 women. The total number of pregnancies of these women was obtained from registered data in SMBR at the time of follow up.

**Fig. 1:**
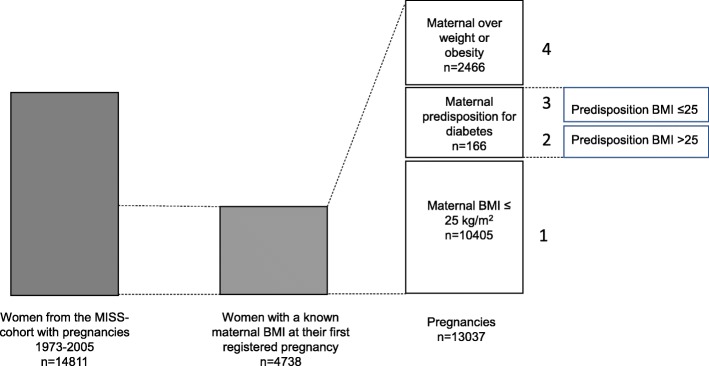
Flowchart of women and pregnancies included in the study. The pregnancies are divided into four different groups according to predisposition to diabetes and BMI

We calculated BMI at admission to the prenatal care unit, at approximately 10-12th week of gestation. The pregnancies were divided into four different groups. The first group consisted of pregnancies in women with pregestational obesity or overweight (BMI ≥ 25 kg/m^2^) at the beginning of that specific pregnancy, but without diabetes at any time. This group (GROUP 1) is hereafter referred to as “overweight” pregnancies (*n* = 2466). Pregnancies of women with “transitory” diabetes reported in SMBR (assumed to be GDM) during **any** of her registered pregnancies (*n* = 66) and pregnancies of women with diabetes reported at follow up (but not during pregnancy) (*n* = 162), were divided into two subgroups according to pregestational BMI, (GROUP 2 - BMI ≥ 25 kg/m^2^; *n* = 75 or GROUP 3 - BMI < 25 kg/m^2^; *n* = 91). GROUP 4 consisted of pregnancies of women with normal weight (BMI < 25 kg/m^2^) who did not have diabetes at any time and this group was used as a reference population (*n* = 10,405) (Fig. 1).

We will hereafter continue to report on data regarding specific pregnancy/ies and not data regarding groups of women. If the woman had multiple pregnancies during the study period and had significant weight change between pregnancies, the pregnancies might be reported in different subgroups depending on BMI. For women who had insufficient data at first registered pregnancy, later pregnancies with complete data could be included in the study.

We calculated parity for each woman from the variables indicating previous pregnancies and the total number of pregnancies in SMBR. In the following analysis of the pregnancies we have included all the pregnancies independent of the parity of the woman. We also did an analysis of only the first registered pregnancy in SMBR. However, this is not equal to the woman’s first pregnancy, since the woman may have had pregnancies prior to 1973, when SMBR started.

We collected data regarding maternal weight at the beginning of each pregnancy, weight-gain during pregnancy (registered in SMBR 1982–1989 and thereafter as a calculated value of weight at delivery minus weight at the beginning of pregnancy), maternal length, smoking habits, caesarean section, post-natal complications, birth weight of the infant and Apgar score at 1, 5 and 10 min. Apgar score rates the pulse, irritability, colour, respiration and activity of the infant and is an indicator of the well-being of the infant. The maximum score is 10. A low Apgar score was defined as an Apgar score of ≤7. The definition of macrosomia is a birth weight ≥ 4000 g. Data regarding large for Gestational age (LGA) (2SD above mean) was retrieved from data reported in the SMBR.

### Statistical analyses

Statistical analyses were performed using the SPSS version 17.0 and 22 statistical software for PC, (SPSS Inc., Chicago, Illinois). Median and range are reported for non-normally distributed variables such as BMI, birth weight, gestational length, gestational weight gain (GWG) and maternal age. Frequencies in percent are reported for categorical variables such as smoking, caesarean section, low Apgar score and instrumental delivery.

For comparison between the groups Mann Whitney U-test (BMI, gestational weight gain, gestational length, maternal age, birth weight,) were used. For comparison of frequencies (smoking, caesarean section, LGA, low Apgar score) Chi 2 test was performed*.* Chi 2 test was replaced by Fischer’s exact test if any observation in any cell was below five. Correlations were tested with Pearson’s and Spearman’s correlation test depending om normal- and non-normal distribution. Multiple regression analyses were done to estimate the impact of different factors on the risk for caesarean section and macrosomia. BMI in the multiple regression analysis was presented in 5 kg/m^2^ intervals.

Ethical Approval**:** This study was conducted in accordance with the Helsinki Declaration. The study was approved by the Ethical Review Board at Lund University, Box 133, S- 221 00 Lund, Sweden. All patients provided written consent at the time of inclusion in the study. Information about additional data being collected was provided through the major newspaper in southern Sweden.

## Results

### Women

The parity for total number of women included in this study was a mean of 2.1 (Median 2.0 (Range 1–10)) pregnancies per woman. The mean BMI for the whole cohort was 22.2 kg/m^2^.

There were 712 women who had pregestational overweight at their first registered pregnancy but without diabetes at any time. Their median age at first pregnancy was 29 [17–44] years and their median BMI was 26.9 [25–41] kg/m^2^. Their median parity was 2 [1–9] children/woman (mean 2.0).

There was 3904 women with normal weight at the first pregnancy. Their median age at first registered pregnancy was 28.0 [17–49] years and their median BMI at first pregnancy was 21.2 (13.1–25) kg/m^2^. These women had a median of 2 [1–8] children/woman (mean 2.2) during the study period. The parity was significantly lower in the group of overweight women compared to women with BMI ≤ 25 kg/m^2^ (*p* < 0.001).

Predisposition to diabetes was observed in 228 women. They had a total of 445 pregnancies with a predisposition to diabetes. However, a pregestational BMI was only available in 166 pregnancies. The median BMI were 23.8 kg/m^2^ and the mean gestational weight gain was 13.3 kg, during these pregnancies. The mean gestational length was 39.6 (median 39.9) weeks and the mean weight of the infant was 3602 g. LGA was registered in 46 of 440 pregnancies (10.5%) and the frequency of caesarean section was 15.2% (*n* = 69/445).

The women with a predisposition to diabetes did not statistically differ regarding parity, compared to women without a predisposition in a comparable BMI-group (p = ns, and p = ns; respectively).

There was no difference in neither maternal weight at beginning of pregnancy (62.3 vs. 60.9 kg; ns) nor weight at delivery (75.9 vs 75.5 kg; ns) in the women with a registered BMI compared to women who did not have a registered BMI.

### Pregnancies

If the woman had multiple pregnancies, all pregnancies was registered and classified according to current pregestational BMI. The pregnancies in the group of women with a predisposition to diabetes were divided into subgroups according to the pregestational BMI. In 75 pregnancies the women had a BMI ≥25 kg/m^2^ and in 91 pregnancies the women had a BMI < 25 kg/m^2^.

The different data divided by subgroups are reported in Table 1.

**Table 1:** Characteristics of pregnancies according to subgroups

	Overweight GROUP 1	Predisp to diabetes over weight GROUP 2	Predisp to diabetes normal weight GROUP 3	Controls GROUP 4	*p*-value Over weight vs. Controls	*P*-value predisp to diabetes over weight vs. Over weight	*P*-value predisp to diabetes normal weight vs Controls
N	2466	75	91	10,405			
Frequency LGA^a^ (%)	7.1	23.3	11.6	2.9	**< 0.001**	**< 0.001**	**< 0.001**
Macrosomia (%)	28.5	34.7	28.6	17.6	**< 0.001**	0.473	**0.012**
Frequency caesarean section^a^ (%)	14.9	24.0	14.3	10.1	**< 0.001**	**0.031**	0.2
Low Apgar^b^ 1 min (%)	8.4	14.9	6.6	6.2	**< 0.001**	**0.05**	0.9
Low Apgar^b^ 5 min (%)	2.1	1.4	2.2	1.5	0.4	0.6	0.6
Low Apgar^b^ 10 min (%)	0.9	1.7	1.4	0.6	0.9	0.6	0.4
Birth weight (g)	3689 (570–5680)	3760 (870–5800)	3595 (1950–5250)	3530 (540–5820)	**< 0.001**	0.165	**0.042**
Gestational length (weeks)	40.1 (25–44)	39.6 (26–42)	39.7 (34–42)	40 (24–44)	0.12	**0.001**	**0.08**
BMI Median	27.1 (25.0–45.4)	28.3 (25.1–44.7)	21.5 (16.9–24.9)	21.5 (13.2–25.0)	**< 0.001**	**< 0.001**	0.45
Median gestational weight gain (kg)	13.0 (−7–30)	11.0 (0–24)	14.5 (5–27)	14.0 (−2–38)	**< 0.001**	**0.03**	0.297
Median age of mother all pregnancies (yrs)	32 (17–46)	32 (19–42)	29 (19–41)	30 (17–49)	**< 0.001**	0.827	0.552
Smoking (%)	22.0/2369	26.1/69	29.1/86	22.0/9865	0.96	0.41	0.12

^a^Data regarding LGA, caesarean section, forceps delivery and vacuum extraction were collected from SMBR

^b^A low Apgar score was defined as ≤7

### Caesarean section

The highest frequency of Caesarean section was seen in the group with both pregestational overweight and a predisposition to diabetes. There was a significantly higher frequency of caesarean section in this group compared to pregnancies with only overweight. But there was no difference when comparing the frequency of caesarean section in pregnancies with a predisposition to diabetes and normal weight, to pregnancies in normal weight controls (Table 1). Higher BMI and higher maternal age were the dominating risk factors for caesarean section. The highest OR for caesarean section was seen in the group with nulliparity. A predisposition for diabetes did not significantly increase the risk of caesarean section (Table 2).

**Table 2:** Risk factors for Caesarean Section

	Variable	OR	CI 95%	*p*-value
*n* = 12,509	BMI interval	1.2	1.2–1.4	< 0.001
	Predisposition to diabetes	1.5	1.0–2.3	0.083
	Nulliparity	1.6	1.4–1.8	< 0.001
	Maternal age	1.1	1.06–1.08	< 0.001

### Macrosomia and LGA and birth weight

The highest frequency of LGA was found in the group with a predisposition to diabetes and overweight, and this was significantly higher than in the group who were only overweight. Even in the group with normal pregestational BMI but with a predisposition to diabetes there was a higher frequency of LGA compared to the control group. Birthweight was significantly higher in the group of pregnancies with predisposition to diabetes and normal weight compared to pregnancies in women in the control group. (Table 1). In a regression analysis BMI was the dominating risk factor for macrosomia, along with a predisposition for diabetes. Nulliparous women had a negative risk for macrosomia (Table 3).

**Table 3:** Risk factors for Macrosomia

	Variable	OR	95% CI	*p*-value
*n* = 12,466	BMI interval	1.5	1.45–1.65	< 0.001
	Predisposition for diabetes	1.5	1.07–2.15	0.020
	Nulliparity	0.65	0.59–0.72	< 0.001
	Maternal age	1.0	0.995–1.015	0.31

### Gestational length

The gestational length was significantly shorter in the group of pregnancies with overweight and with diabetes later in life compared to gestational length in pregnancies in the group of women who were only overweight (Table 1).

### Maternal weight and gestational weight gain

**Fig. 2:**
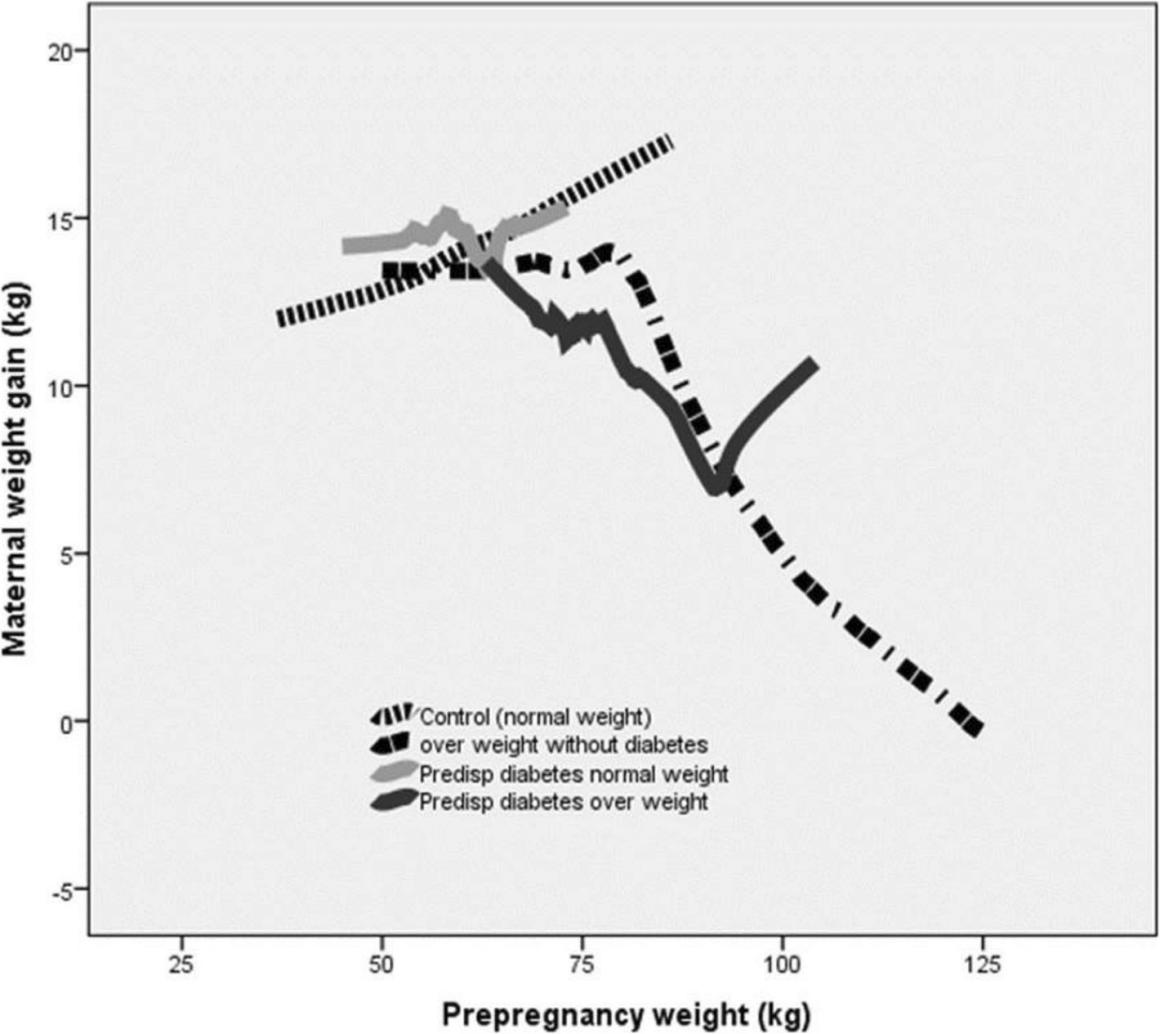
Weight gain during pregnancy was inversely correlated to the pre-pregnancy weight among both overweight women and among the overweight women with a predisposition to diabetes (r = −0.23; *P* < 0.001 and r = −0.30; *P* = 0.04, respectively), while there was a positive correlation in the control group (r = 0.16; *P* < 0.001)

Overweight women with predisposition for diabetes had significantly lower maternal weight gain compared to overweight women without diabetes later in life who, in their turn, had lower weight gain compared to controls. In the group of pregnancies with overweight and a predisposition to diabetes we noted a significantly higher BMI, but a lower gestational weight gain compared to pregnancies with only maternal overweight. Normal weight women with a predisposition to diabetes had similar GWG as women in the control group (Table 1). The weight gain was inversely correlated to the pre-pregnancy weight among both overweight pregnancies and among the overweight pregnancies with a predisposition to diabetes (r = − 0.23; *p* < 0.001 and r = − 0.30; *p* = 0.04, respectively), while there was a positive correlation in the control group (r = 0.16; *P* < 0.001). There was no significant correlation in the normal weight group with predisposition to diabetes (Fig. 2).

### Maternal age

There was no significant difference in maternal age during pregnancy in the groups with a predisposition to diabetes, compared to GROUP 1 and GROUP 4, respectively. In the pregnancies with obesity there was a significant higher maternal age than in the control group (Table 1).

### Apgar scores

There was a higher frequency of low Apgar score at 1 min among infants born to overweight women with a predisposition to diabetes compared to infants born to overweight women without a predisposition to diabetes (Table 1).

### First registered pregnancy

In a subgroup of data only the woman’s first registered pregnancy was included, and the number of cases is therefore reduced. The results are presented in Table 4. The median BMI and the frequency of LGA was significantly higher in the pregnancies with a predisposition to diabetes with normal weight compared to pregnancies with normal maternal weight without predisposition to diabetes. Similar to previous analysis with all pregnancies included, there was a significantly higher frequency of low Apgar scores at 1 min in the group with pregestational over weight and a predisposition to diabetes, compared to the group who were only over weight. The birth weight was significantly higher in the pregnancies with a predisposition to diabetes and normal weight, compared to normal weight controls. When analysing the first registered pregnancy, the frequency of macrosomia in pregnancies with a predisposition for diabetes was numerical higher but did not reach statistical significance.

**Table 4:** In a separate analysis only the first registered pregnancy of the woman was included

	Overweight	Predisp to diabetes over weight	Predisp to diabetes normal weight	Controls	*p*-value Over weight vs. Controls	*p*-value predisp to diabetes over weight vs. Over weight	*p*-value predisp to diabetes normal weight vs Controls
N	723	25	29	3961			
Frequency LGA^a^ (%)	3.9 (27/696)	12.0 (3/25)	11.1 (3/27)	1.3 (49/3839)	**< 0.001**	0.081	**0.005**
Frequency Macrosomia (%)	21.2 (153/723))	24.0 (6/25)	20.7 (6/29)	11.6 (460/3961)	**< 0.001**	0.623	0.125
Frequency ceasarean section^a^ (%)	18.3 (132/723)	16.0 (4/25)	17.2 (5/29)	12.0 (476/3961)	**< 0.001**	0.774	0.386
Low Apgar^b^ 1 min (%)	11.0 (79/720)	25.0 (6/24)	10.3 (3/29)	9.1 (359/3924)	0.124	**0.034**	0.745
Low Apgar^b^ 5 min (%)	3.8 (27/718)	4.2 (1/24)	3.4 (1/29)	2.3 (91/3906)	**0.025**	0.609	0.498
Low Apgar^b^ 10 min (%)	1.3 (8/603)	5.0 (1/20)	0 (0/29)	0.9 (29/3122)	0.367	0.256	1.0
Birth weight Median (Range) (g)	3520 (770–5440)	3690 (870–5800)	3630 (2650–5000)	3430 (540–5240)	**< 0.001**	0.270	**0.020**
	*n* = 722	*n* = 25	*n* = 29	*n* = 3940			
Gestational length Median (Range) (weeks)	40.1 (25.0–43.3)	39.7 (25.7–41.7)	40.0 (35.4–42.3)	40.0 (25.4–44.4)	0.105	0.148	0.987
	*n* = 722	*n* = 25	*n* = 29	*n* = 3955			
BMI Median (Range) Kg/m^2^	27.0 (25.0–40.5)	28.7 (25.3–37.0)	21.0 (18.0–23.7)	21.2 (13.2–25.0)	**< 0.001**	**0.003**	0.328
	*n* = 723	*n* = 25	*n* = 29	*n* = 3961			
Median gestational weight gain	14.0(−2–30)	12.0 (3–24)	15.0 (5–22)	14.0 (0–35)	0.197	0.147	0.837
	*n* = 477	*n* = 19	*n* = 21	*n* = 3367			
Median age of mother first pregnancy (yrs)	29 (17–44)	28 (19–41)	27 (19–40)	28 (17–49	**< 0.001**	0.607	0.946
	*n* = 723	*n* = 25	*n* = 29	*n* = 3961			
Smoking (%)	24.6	28.6	33.3	22.6	0.26	0.33	0.32

^a^Data regarding LGA, caesarean section, forceps delivery and vacuum extraction were collected from SMBR

^b^A low Apgar score was defined as ≤7

## Discussion

Women with a predisposition to diabetes had an increased risk to deliver macrosomic infants. If the woman at the same time was overweight at the start of pregnancy, there was a even higher frequency of LGA. Women with predisposition to diabetes had a more than 3 times higher frequency of LGA than women without predisposition to diabetes. If the woman had both a predisposition to diabetes and overweight there was an almost 8 times higher frequency of LGA, compared to normal weight controls. These women also had a higher frequency of Low Apgar score and caesarean section.

During recent years the negative influence of overweight and obesity on pregnancy outcome has come into focus. The increasing frequency of overweight and obesity especially in low educated young women in child bearing ages contributes to a growing health problem [25]. Poor socioeconomic factors and an unhealthy life style among women also have implication for the next generation since children raised in these families show a higher prevalence of obesity [26].

A major weakness of this study is the lack of data regarding pregestational BMI. However, there was no difference in neither maternal weight at beginning of pregnancy nor weight at delivery in the groups with or without BMI registered at first pregnancy. Therefore, we do not suspect that this is anything but random. The women with registered BMI at first pregnancy are significantly older (28.4 vs. 26.9; *p* < 0.001). The implication of this is uncertain. Another weakness is the absence of p-glucose values during pregnancy and the lack of specification of diabetes type in the SMBR. General screening for gestational diabetes was not performed at the time when these women were pregnant, explaining the extremely low frequency of diagnosed GDM in the register. The low frequency of reported diabetes during pregnancy suggests that GDM has been overlooked in many of the women of our cohort. Presently a general screening with Oral Glucose Tolerance Test (OGTT) identifies GDM in 2–3% of all pregnancies in our region [27] and follow up of these women have shown that as many as 30% of women with GDM develop diabetes or IGT within 2 years of follow up [28]. There is therefore good clinical evidence for the assumption that many of the women who developed diabetes later in life have had hyperglycaemia and/or GDM during pregnancies earlier in life.

In SMBR the first registered pregnancy may not be the woman’s first pregnancy, since she may have had pregnancies before the register started in 1973. Since this might influence the maternal weight at the start of following pregnancy, we have chosen to analyse the outcome of pregnancy based on current BMI at the start of pregnancy, rather than the BMI at the start of the study.

It is previously known that multiple pregnancies increase the risk of developing type 2 diabetes [29]. Weight retention and increasing overweight between pregnancies may partly explain this. Women with overweight had a significant lower parity than normal weight women in this study. There was no difference in frequency of smoking between groups. However, other socioeconomic factors were not analysed in this study.

The major strength of this study is that it is a large cohort, representing the general population, with a long follow up time. The lack of glucose-values or OGTT may also be considered a strength in this study since glucose values were unknown to the woman and the medical caregivers who subsequently did not routinely give any special treatment or intervention that could bias the outcome. The natural course could therefore be studied.

### Caesarean section

Both a high maternal BMI and maternal age are the main risk factors for caesarean section [30]. A low pregnancy weight gain among obese women can reduce an otherwise high risk for caesarean section [31]. In this cohort, the highest risk for caesarean section was seen in the group with overweight and a predisposition for diabetes. This group had the lowest GWG, but still above IOM recommendations. In this study the overweight women were older. However, in the group of pregnancies with overweight and a predisposition for diabetes, there was no difference in maternal age that could explain the higher frequency of caesarean section. The reason for caesarean section could not be examined in this study. The explanation for shorter gestational length in the group of women with overweight and predisposition to diabetes is also unclear. It can be speculated that there were medical indications for interruption of pregnancy, induction of labour or reasons for caesarean section. It is also conceivably that preterm labour was more common in the group of overweight women with predisposition to diabetes. In a multivariate regression analysis BMI and nulliparity seems to be the dominating risk factors for caesarean section which is consistent with previous studies [32, 33]. Having a predisposition for diabetes did not significantly increase the risk of caesarean section.

The lower Apgar scores at 1 min in pregnancies with high maternal BMI could indicate a higher frequency of complications during delivery. It may also be a result of the higher frequency of caesarean section. There was a significant shorter gestational length in the pregnancies with a predisposition to diabetes. The reason for this is not studied and merits further investigation. Speculative, it might be caused by induction of labour, planned or emergency caesarean section due to a or a medical issue in the health of the mother or due to a macrosomic infant. It might also be due to planned or emergency caesarean section. A higher frequency of a low Apgar score in this group might be consistent with this.

### Macrosomia and Birthweight

The frequency of LGA was consistently higher in the group of women who were overweight at the start of pregnancy or had a predisposition for diabetes regardless of prepregnancy BMI. These finding were consistent when analysing only the first registered pregnancy. Risk factors for macrosomia includes a predisposition for diabetes. It is possible that the higher birthweight was caused by higher blood glucose in the mother, within normal range or an undiagnosed GDM. However, this study has not analysed glucose values during pregnancy.

### Gestational weight gain

This study does not support that high GWG may contribute to the higher frequency of LGA and caesarean section in the women with overweight and a predisposition for diabetes. The women in this group had the lowest GWG. Similar correlations have previously been reported among overweight women without diabetes [10, 34–36]. In this study a lower GWG might be explained by the shorter gestational length. It is unlikely that shorter gestational length was due to intervention of a known GDM, since the reported number of individual pregnancies with reported transitory diabetes is only 25 in SMBR. Instead it may partly be explained by a higher frequency of caesarean section in the pregnancies with a predisposition to diabetes.

Overall, the women with overweight were older and it is possible that these women more often had multiple pregnancies and weight retention between pregnancies. In this material we could not confirm that the women who were overweight at the time of their first registered pregnancy had a higher parity.

The recommendations from IOM regarding GWG should be observed and this will certainly improve outcome in some pregnancies. However, the remaining and larger problem is the high frequency of young women who are overweight at the start a pregnancy since prepregnancy BMI is a stronger predictor of caesarean delivery [24, 43–47]. This is consistent with the findings in our study where the group with the highest BMI had the lowest weight gain, but despite this, the highest frequency of caesarean section.

In this study we have identified women who developed diabetes after pregnancy and we assume that these women, with a predisposition to diabetes, could have been identified with an OGTT during pregnancy but this is only a surmise since the study lack measurements of glucose or OGTT tests. If obese women with this metabolic disturbance would be possible to identify during pregnancy, intensified prenatal, and perhaps postnatal care could be offered to them. The way to identify these women could be either with a conventional OGTT or by some metabolic marker for metabolic distress or cardiovascular disease. Several such markers have already been described and others are under investigation [48–50] The high number of overweight women could be further classified into women with benign type of overweight and women with a hazardous overweight and a high risk for future diabetes and cardiovascular disease. Thus, enabling resources and interventions to be focused on the women who need it the most.

## Conclusions

In this large retrospective study of a large cohort, we found that there was an increased risk of complications during delivery for women who were overweight or obese at the start of pregnancy. The risk of complications in pregnancy was the highest in the group who was overweight and at the same time had a predisposition for developing diabetes later in life*.* Screening for gestational diabetes, and even prediabetes or other metabolic risk factors, among pregnant women with obesity, may enable health care units to identify women with the highest risk for adverse outcome of pregnancy and the highest risk of developing diabetes in the future.

## Data Availability

The datasets generated and analysed during the current study are not publicly available due to patient integrity and patient-doctor confidentiality. The data that support the findings of this study are available on request from the corresponding author [UM]. The data are not publicly available due to legal regulations from the Ethical Board*.*

## Abbreviations

BMI: Body Mass Index
GDM: Gestational Diabetes Mellitus
GWG: Gestational weight gain
LGA: Large for gestational age
OGTT: Oral Glucose Tolerance Test
OR: Odds Ratio
SMBR: Swedish Medical Birth Registry
