# Correction to: Women with a predisposition for diabetes have an increased risk of pregnancy complications, especially in combination with pregestational overweight

**DOI:** 10.1186/s12884-020-2803-8

**Published:** 2020-02-19

**Authors:** Ulrika Moll, Håkan Olsson, Mona Landin-Olsson

**Affiliations:** 1 Department of Endocrinology, Lasarettsgatan 15, Skane University Hospital, S-221 85 Lund, Sweden; 2 Department of Clinical Sciences, Lund University, Lund, Sweden; 3 Departments of Oncology & Pathology and Cancer, Epidemiology, Skane University Hospital, Lund, Sweden

**Correction to: BMC Pregnancy Childbirth (2020) 20:74**

**https://doi.org/10.1186/s12884-020-2741-5**

Following publication of the original article [1], we have been notified by the author that the age of women from the Result section was incorrectly tagged as references.

The references tag for the age of women from the Result section has been removed.

The original article has been corrected.
